# Urbanization and Health Inequity in Sub-Saharan Africa: Examining Public Health and Environmental Crises in Douala, Cameroon

**DOI:** 10.3390/ijerph22081172

**Published:** 2025-07-24

**Authors:** Babette Linda Safougne Djomekui, Chrétien Ngouanet, Warren Smit

**Affiliations:** 1 African Centre for Cities, University of Cape Town, Cape Town 7700, South Africa; warren.smit@uct.ac.za; 2 National Institute for Cartography, Yaoundé P.O. Box 157, Cameroon; chngouanet@yahoo.fr

**Keywords:** urbanization, health inequalities, healthcare access, public health crises, informal settlements, social determinants of health, sub-Saharan Africa

## Abstract

Africa’s rapid urbanization often exceeds the capacity of governments to provide essential services and infrastructure, exacerbating structural inequalities and exposing vulnerable populations to serious health risks. This paper examines the case of Douala, Cameroon, to demonstrate that health inequities in African cities are not simply the result of urban growth but are shaped by spatial inequities, historical legacies, and systemic exclusion. Disadvantaged neighborhoods are particularly impacted, becoming epicenters of health crises. Using a mixed-methods approach combining spatial analysis, household surveys and interviews, the study identifies three key findings: (1) Healthcare services in Douala are unevenly distributed and dominated by private providers, which limits access for low-income residents. (2) Inadequate infrastructure and environmental risks in informal settlements lead to a higher disease burden and an overflow of demand into better-equipped districts, which overwhelms public health centers across the city. (3) This structural mismatch fuels widespread reliance on informal and unregulated care practices. This study positions Douala as a microcosm of broader public health challenges in rapidly urbanizing African cities. It highlights the need for integrated urban planning and health system reforms that address spatial inequalities, strengthen public health infrastructure, and prioritize equity—key principles for achieving the third Sustainable Development Goal (ensuring good health and well-being for all residents) in sub-Saharan Africa.

## 1. Introduction

Urbanization in Africa is occurring at a rapid pace, radically reshaping cities. While this transformation has the potential to drive economic development and improve living conditions, the reality for many African cities is far more complex. Rapid urban expansion often outpaces the ability of governments to provide essential infrastructure and services, leading to significant disparities in accessing basic resources such as healthcare, clean water, and adequate housing [1,2]. These challenges are particularly acute in informal settlements, where residents face increased risks of public health crises and poor health outcomes [3]. A growing proportion of urban residents now live in informal settlements, with limited or no access to basic services and public facilities and with high exposure to severe environmental health challenges [2,4,5,6]. In addition, healthcare systems are struggling to provide adequate and affordable healthcare services. The differential exposure to these challenges, compounded by social and economic vulnerability, exacerbates health inequities, especially in informal settlements [3,7]. The lack of adequate housing, sanitation, and access to healthcare services in these vulnerable areas contributes significantly to health disparities [1,8] and is a major risk factor for infectious diseases [9,10,11].

Douala, the economic capital of Cameroon, exemplifies the complex nature of urbanization in Africa. On the one hand, it is a hub of economic activity and opportunity, drawing individuals from across the country and the central African sub-region who are seeking improved living conditions. On the other hand, the rapid and largely unplanned urban expansion of the city has resulted in severe socio-spatial inequalities, with informal and disadvantaged neighborhoods bearing the brunt of inadequate infrastructure and limited access to healthcare [12]. These neighborhoods are marked by overcrowded living conditions, poor sanitation and a lack of other basic services, which together create a fertile ground for the proliferation of health crises [1,6]. The health inequities observed in Douala are not merely a byproduct of urban growth but are also deeply rooted in historical, socio-political, and economic factors. As has been found in several African cities, colonial legacies have strongly influenced the spatial layout of the city and have contributed towards patterns of segregation that still persist [13,14]. Post-colonial policies have often failed to address these disparities, further entrenching socio-spatial inequalities and limiting access to essential services for the most vulnerable populations. As a result, informal settlements have become epicenters of health crises, ranging from “everyday” risks such as infectious and parasitic diseases to larger-scale crises [5].

Despite the growing awareness of urban health challenges in sub-Saharan Africa, there is not enough empirical and spatial analysis of how rapid urbanization, environmental exposures, and informal health systems intersect to reinforce health inequalities and inequities in sub-Saharan African cities. Furthermore, the spatial mismatch between health infrastructure and the most vulnerable populations is poorly understood, particularly at the intra-urban scale. This study seeks to address this knowledge gap by exploring the following questions:

What socio-economic and environmental factors shape health vulnerability in informal settlements?

How is access to healthcare facilities, both public and private, distributed across health districts in Douala?

What alternative healthcare practices emerge in response to poor access to formal services?

How do these patterns reflect systemic inequalities and contribute to a wider health crisis?

### 1.1. Theoretical and Conceptual Frameworks

**Figure 1:**
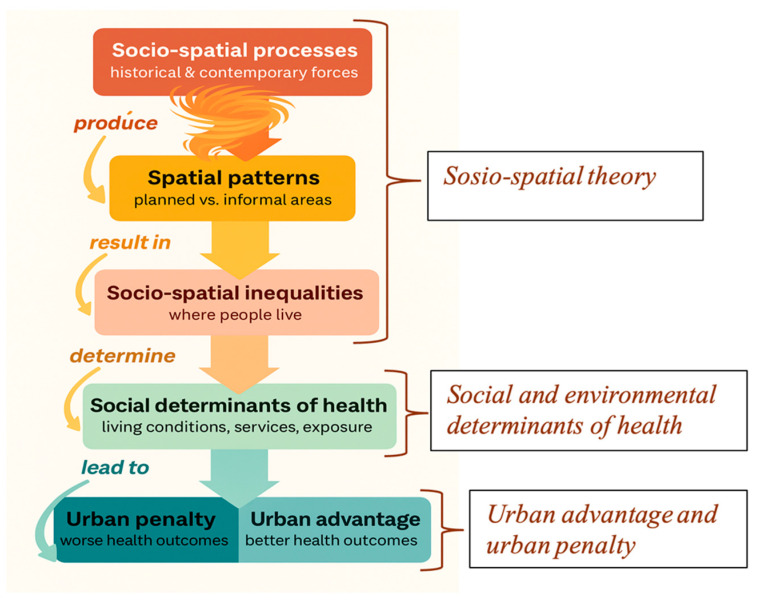
Analytical framework of the study (designed by the authors).

The analytical framework of this study is based on the integration of three key theoretical and conceptual lenses, namely socio-spatial theory, social and environmental determinants of health (SDOH), and the urban health advantage/penalty debate. Together, these approaches provide a critical understanding of how systemic urban inequalities, spatial dynamics, and socio-economic factors interact to influence health outcomes in Douala’s marginalized neighborhoods. They provide a framework to understand why health crises are not randomly distributed across the city but are structurally produced and spatially concentrated (Figure 1).

### 1.2. Socio-Spatial Theory

Socio-spatial theory, first developed by Henri Lefebvre [15] in *The Production of Space*, argues that space is socially produced and not a passive, neutral container of social processes. According to Lefebvre, spatial configurations emerge from social, political, and economic forces, which in turn reinforce particular social relations, inequalities, and power structures. In the urban context, Soja E. [16] further developed the concept of the socio-spatial dialectic, which underscores the mutually constitutive relationship between society and space. Soja argues that the way cities are spatially organized reflects social hierarchies, and these spatial forms then feedback to maintain or challenge social structures.

### 1.3. Social and Environmental Determinants of Health

The World Health Organization (WHO) defines social determinants of health as the non-medical factors that influence health outcomes. These are the conditions in which people are born, grow, work, live, and age, as well as the broader set of forces and systems that shape the conditions of daily life. In addition to social and economic factors, the World Health Organization (WHO) recognizes that the physical environment of individuals and communities is also a key determinant of health. These determinants, or factors that influence health, include the following:

- Income and social status: Higher income and social status correlate with better health. The greater the gap between the richest and poorest people, the greater the differences in health.
- Education: Low levels of education are linked with poor health outcomes.
- Physical environment: Safe water, clean air, healthy workplaces, and safe homes, communities, and roads all contribute to good health.
- Health services: Access to and use of services that prevent and treat disease has a major impact on health.

### 1.4. Urban Health Advantage and Urban Health Penalty

The urban health advantage argument suggests that cities generally offer better access to quality services, infrastructure, and economic opportunities, leading to better health outcomes than rural areas. However, in many African cities, including Douala, this benefit is unequally distributed and increasingly counterbalanced by the urban health penalty effect. The urban health penalty refers to the fact that vulnerable populations, such as those living in informal settlements, experience worse health outcomes than their rural counterparts due to overcrowding, poor living conditions, environmental hazards, and exclusion from essential services.

## 2. Materials and Method

### 2.1. The Study Area

**Figure 2:**
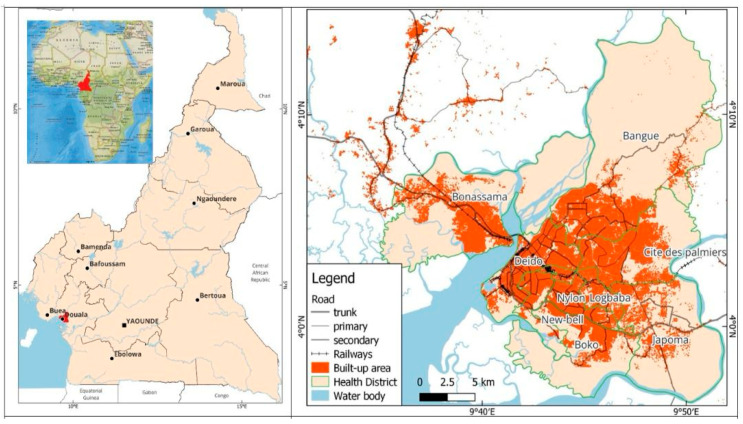
Location of the study area (city of Douala).

The study was carried out in Douala, Cameroon’s economic capital and business hub (Figure 2). Douala is a port city, located on the Wouri estuary, 30 km from the Atlantic Ocean, at the heart of the Gulf of Guinea. It has an estimated population of more than 4 million people.

The city of Douala is a pertinent case study of the complex challenges facing African cities. It is characterized by rapid and largely informal urbanization, significant socio-spatial segregation, growing environmental challenges, and a public health crisis that is particularly acute in the most disadvantaged neighborhoods.

Administratively, the city of Douala is the capital of the Littoral Region, which is the most densely populated of the ten regions of Cameroon. Although Douala accounts for less than 5% of the region’s territory, it accounts for 76% of its total population, and 82% of its urban population [17].

### 2.2. Understanding the Study Setting

#### 2.2.1. Structure of the Healthcare System in Cameroon

The health system in Cameroon is organized into three levels: (a) the central level (policy and strategy development undertaken by the Ministry of Public Health); (b) the intermediate level (regional delegations overseeing regional hospitals); (c) the peripheral level made of health districts and health areas [18]. Following the healthcare map of Cameroon, the city of Douala is structured into eight health districts. Health districts serve as the key operational units for Cameroon’s decentralized healthcare delivery system, ensuring that resources and services are allocated closer to communities [18]. These districts are designed to bridge national healthcare policy and local implementation. Each district is structured to include various levels of centers within the healthcare pyramid (Table 1), making it a natural lens for analyzing healthcare delivery, resource distribution, and accessibility. By focusing on health districts, the analysis can identify disparities in infrastructure, service availability, and gaps in meeting the population’s healthcare needs. Health districts also reflect the reality of healthcare access for most residents, as patients typically seek care within their district of residence. This approach helps to explain local healthcare challenges, such as overcrowding in public facilities, the dominance of private providers, and inequities between urban and peri-urban areas. Additionally, analyzing health districts aligns with Cameroon’s national health policy, which decentralizes healthcare delivery to districts in order to improve efficiency and accessibility.

The healthcare system in Cameroon is structured as a pyramid with three tiers of services, distributed across six categories of healthcare facilities ranging from primary care to specialized interventions. The structure includes integrated health centers at the community level, offering basic services such as vaccinations and consultations. At higher levels, district medical centers and district hospitals provide more comprehensive care, including general medicine, obstetrics, and surgery. The top tier consists of regional hospitals, central hospitals, and general/reference hospitals, which handle specialized and complex medical cases (Table 1). This system integrates public facilities run by the state and private facilities that complement the public system. However, it is important to note that such private centers are often financially inaccessible to lower-income populations due to high costs.

#### 2.2.2. Understanding Douala’s Urban Growth Through the Socio-Spatial Dialectic: How Inequality Is Produced in Space

This section draws on Lefebvre’s concept of the production of space and Soja’s socio-spatial dialectic to understand how Douala’s urban landscape has become a site of long-standing spatial inequalities. The urban growth of Douala was not a random process, rather it was continuously shaped by historical, political, and economic forces that produced and organized space in a way that reflected and reinforced social hierarchies. By tracing the city’s evolution from the colonial era to the present day, this study reveals how various stages of planning, governance, and informal expansion have resulted in a profoundly imbalanced urban landscape where inequalities are embedded in the fabric of the city.

- Historical Legacies of Socio-Spatial Inequities in Douala

The urbanization process and socio-spatial dynamics of Douala following Cameroon’s independence provide crucial insights into the city’s persistent vulnerability to natural hazards [19,20]. This transformation can be analyzed in different phases, each shaping the socio-spatial structure of the city. These phases trace the evolution of urban growth, from the colonial era to the present, illustrating how socio-economic and political forces have influenced the city’s development.

- Colonial Era (Pre-1960): Creation of the colonial city

During the colonial period, Douala’s urban growth was defined by the strategic choice of its location, particularly its establishment as a port city. German settlers initiated the first development of the port in 1881, marking the beginning of the city’s transformation into a key commercial hub. The establishment of trading posts along the coast was followed by the expropriation of indigenous lands to accommodate European settlements and resettlement villages. These actions laid the foundations for the first expressions of socio-spatial segregation and inequity, as the “European city” and the native resettlement villages were intentionally separated by a “free zone”. Roads were also constructed to facilitate commercial activities, reinforcing the city’s role as a trading center. This phase of urbanization marked the roots of spatial inequalities being solidified, particularly through land speculation, which increased with the arrival of French settlers and their continued development of the port and industries.

- The Planned City: “The Thirty Glorious Years” (1960–1988)

After Cameroon gained independence, the period known as the “Thirty Glorious Years” marked a phase of state-driven urban planning. The Cameroonian government adopted a welfare state model, focusing on planned, self-sufficient urban development. This period was characterized by the implementation of development plans initiated by the colonial administration. Prominent among these was the Orian plan, alongside the launch of five-year plans that sought to promote infrastructural development and social housing through initiatives like the Cameroon Real Estate Company. This phase witnessed the formation of planned neighborhoods and formal settlements. However, as urban population growth accelerated, spatial demand surged and environmental risks became an increasing threat to the city. These challenges were partially addressed in the fifth five-year plan (1981–1986), though they continued to grow, signaling the limitations of planned urbanism in addressing the needs of a rapidly expanding population.

- Formation of the Informal City (1987 to Early 2000s)

The economic crisis that began in 1987 significantly impacted the state’s ability to manage urban growth. The implementation of Structural Adjustment Policies (SAPs) led to the national government’s withdrawal from centralized urban planning, thereby paving the way for a decentralization of governance. The creation of the Douala Urban Community, along with the failure of the sixth five-year plan, marked a turning point in urban development. This period was marked by a disproportionate spatial expansion as migrants and low-income residents, unable to afford living in the city center, began settling in high-risk peripheral areas. These informal settlements grew rapidly, driven by both migration and land speculation. Informality became a key strategy for survival, with many households adapting to life in areas lacking basic infrastructure. The shift from economic liberalization to the unregulated expansion of the city led to a deepening of spatial inequality, as the wealthier residents remained in the planned city center, while the poor were “pushed” to the periphery.

- Attempts to Regain Control of the City’s Development (2000s to Present)

Since the 2000s, there has been a renewed political will to address the chaos and disorganization in Douala’s urban growth. The government has made efforts to implement the international commitments outlined in Agenda 21 for Douala, including the creation of urban planning strategies and the development of urban frameworks such as the Land-Use Plan and the Urban Development Plan. Despite these efforts, urban sprawl continues, and tensions between local authorities and residents have risen, particularly concerning demolitions and evictions in areas deemed high-risk. Social conflicts have emerged, as community resistance persists against what is perceived as the unjust treatment of informal residents. Furthermore, the government is now focusing on integrating climate considerations into urban planning as part of broader attempts to address environmental vulnerabilities and climate-related risks.

- Rapid Spatial Expansion and Unplanned Occupation of Flood-Prone Areas

**Figure 3:**
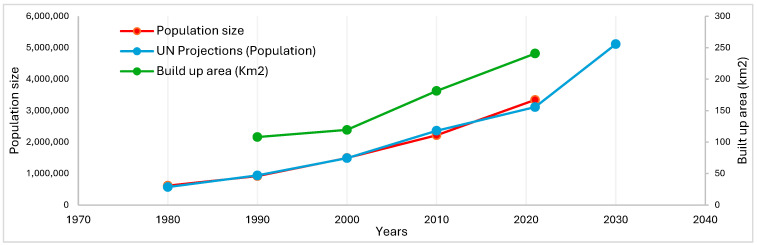
Population growth and urban footprint trends in Douala since 1980. Source: Statistical Yearbook for the Littoral Region, National Institute of Statistics of Cameroon, UN-Habitat.

Over the past 30 years, the urban footprint of Douala has more than doubled from 108 km^2^ to 240 km^2^ [21]. If the current trends in population growth and urban expansion persist, an additional 324 km^2^ will be required to accommodate 1.3 million new residents by 2030 (Figure 3).

As Douala’s population has expanded, the city has undergone rapid and often unregulated spatial growth, with informal settlements increasingly encroaching upon environmentally sensitive zones such as mangroves and floodplains [9,22]. The unplanned occupation of these ecologically fragile areas has heightened residents’ vulnerability to natural hazards, particularly flooding, and associated public health risks. These high-risk zones have evolved into “at-risk neighborhoods,” where environmental challenges are compounded by the absence of essential urban infrastructure and services. Populations residing in these neighborhoods, typically composed of low-income households and rural migrants, have to construct informal housing on unstable land, further exacerbating environmental degradation and increasing their susceptibility to climate-induced hazards.

### 2.3. Data Collection

To investigate complex socially constructed health inequities in Douala, the study used a mixed-methods approach, combining spatial analysis, field surveys, and interviews. Each method directly supports the research questions, triangulating quantitative service access patterns with qualitative lived experiences to unpack health inequities in Douala.

#### 2.3.1. The Fieldwork: Primary Data Collection

The research problem addressed in this paper is one aspect of the broader research questions investigated in the fieldwork that this article is based on. The primary data collected during the fieldwork provided contextual insights and relevant statistical information upon which the present study is built. The fieldwork specifically provided insight into living conditions, healthcare demand, and accessibility, as well as the availability of informal healthcare alternatives. Prior to any engagements with the respondents, one or more preliminary meeting was held with the mayor of the relevant subdivision, neighborhood chiefs, and block leaders. These meetings were held to present the research objectives, share our intention to survey households, and obtain their approval. This was performed to gain the residents’ acceptance of the study. The research purposes and objectives were also verbally explained to all respondents, and explicit verbal consent was obtained before commencing the survey or interview. The Boko health district was selected for data collection as it strongly meets the criteria of a low-income, high-risk district, due to its large number of densely populated informal settlements located in flood-prone areas. This makes it one of the city’s most hazardous zones. In addition, observations were made throughout the city to analyze the broader context. The fieldwork also involved examining the consultation registers of three health centers in the Boko district. This was undertaken in order to gain insights into healthcare demand and disease burden, and to correlate this information with that gathered through the household survey.

- *On-site data collection via questionnaire survey*

**Figure 4:**
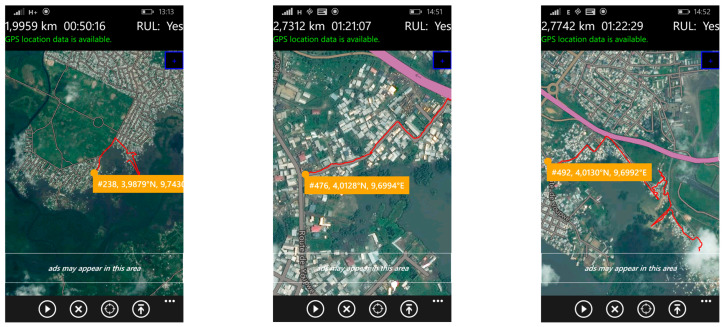
Examples of route traces (red lines) using a GPS during the field work.

The questionnaire surveys were conducted with 260 households in the Boko health district, selected through systematic random sampling. A live GPS tracking system on mobile phones was used to navigate along streets and footpaths serving residential areas, as illustrated in Figure 4. During the pre-survey phase, satellite imagery and non-participant observations were used alongside transect walks to develop an understanding of the area’s spatial characteristics. This preliminary work enabled the targeted health district to be divided into four zones and twelve neighborhoods. These spatial subdivisions were then used to guide the distribution of questionnaires, streamline fieldwork operations, and support spatial monitoring throughout the data collection process.

The survey unit used in this study was the household. A questionnaire was administered to each household selected in our sample on a door-to-door basis, with the head of the household present at the time of the survey. The field visits were all organized in the same way, i.e., a full day of data collection and a systematic consistency check at the end of each day. The questionnaire was designed to gather information on the socio-economic characteristics of the target population. It also helped to collect data on the influence of cultural and identity factors on how space is occupied and managed, as well as on health-related choices and decisions. The questionnaires contained a mix of closed questions (yes/no, evaluation scales, and tick boxes) and open-ended questions, which allowed respondents to provide additional detail to help gather more information on context and perception.

- *On-site interviews*

The interviews were mainly face to face. A total of 60 participants took part, comprising 35 individual semi-structured interviews and 25 people in focus groups. Data from these discussions was primarily collected through notetaking and, where respondents permitted, by recording.

The interview guides were semi-structured to allow participants to express their views freely, while focusing the discussion on predefined themes. This structure facilitated the interviewer’s active but neutral participation through follow-up questions and rephrasing. A total of 35 participants were involved in the semi-structured interviews, as follows: Local representatives (mayor, neighborhood heads, City of Douala staff): 15 participants. Religious and community leaders: 5 participants. Resource persons (heads of households selected based on their seniority in the neighborhood and block leaders): 15 participants.

Five focus groups involving 25 participants in total were held, with group sizes ranging from 4 to 6 participants (two groups of 4, two groups of 6, and one group of 5) with at least 2 women per group. This method enables in-depth, small-group discussions to explore perceptions, opinions, and experiences related to the overall research topic of the doctoral work, which included information on health accessibility, health vulnerabilities, and the existing healthcare alternatives used.

#### 2.3.2. Secondary Data Collection

- Data on urbanization trends and the urban footprint of Douala were collected from various sources, including scholarly publications, national reports, and statistics from United Nations agencies. Additionally, the land-cover dynamics were derived from an analysis of satellite imagery using data from the Landsat 4 TM sensor (1986), Landsat 7 ETM+ sensor (2001), and Land-sat 9 OLI sensor (2022).
- Data on health indicators (especially healthcare distribution, type of healthcare (public/private), and health demand) were collected from the District Health Information System, 2nd version (DHIS2) database for the city of Douala and from the DHIS2 officials’ report at the national level, as well as national and regional reports from the public health department [17,18,23,24,25,26].

### 2.4. Data Analysis

Data analysis was carried out using descriptive statistics. Indicators such as frequencies and percentages were calculated to summarize and present the main characteristics of the variables studied. These analyses were used to draw up a general portrait of the survey population and to identify trends and distributions relating to the main variables of interest using Excel and SPSS software version 31.0.0.0 (117). The results were presented in the form of tables and graphs to facilitate their interpretation. In addition, the statistical analysis of the spatialized health data consisted initially of calculating ratios to produce two important health variables for each disease—the prevalence (number of patients out of the district population) and the case-fatality rate (number of deaths out of the number of cases diagnosed)—by applying the following equations in the Excel spreadsheet:

(1) $$Prevalence\;\left(\%\right)\;=\;100\;\times \;\frac{Number\;of\;Cases}{Total\;population}$$

(2) $$Fatality\;rate\;\left(‰\right)=1000\times \frac{Number\;of\;deaths}{Number\;of\;Cases}$$

Thematic mapping was then used to highlight the spatial distribution of health variables calculated by health district. The choice of representation technique depended on the nature of the variables (quantitative stock or intensity). The variables mapped at the health district level include the following:

- The average number of people per public health center;
- The typology of health centers (public and private);
- The average number of patients per year per illness, for cholera, gastroenteritis, and typhoid;
- The case-fatality rate for certain environmental diseases;
- The population density per health district;
- The land cover dynamics data were produced by processing satellite images from the Landsat 4 TM sensor from 1986, Landsat 7 ETM+ sensor from 2001, and Landsat 9 Oli sensor from 2022. The Normalized Difference Built-up Index (NDBI) was used to extract built-up areas from Landsat images. This is a spectral index commonly used to identify built-up areas on satellite images. It uses the reflective properties of built-up areas in the near infrared (NIR) and shortwave infrared (SWIR) bands to differentiate them from other types of land cover.

(3) $$NDBI=\frac{\left(SWIR-NIR\right)}{(SWIR+NIR)\;}$$

Spatialized data was processed in QGIS, a free and open-source geographic information system (GIS) software version 3.34.11. This software was specifically used in this research for spatial data visualisation, NDVI calculation, and map editing.

## 3. Results

### 3.1. Healthcare Distribution and Access

The issue of health provision in the city of Douala, Cameroon, is a complex one that reflects the challenges faced by urban populations in terms of access to health services in major cities in sub-Saharan Africa. The city of Douala has 17% of all the country’s health facilities and is home to 76% of the health facilities in the Littoral Region. However, this abundance of health services does not guarantee that they are easily accessible to all households, regardless of their living standard and the area in which they live. Also, it does not guarantee that they are of good quality.

#### 3.1.1. Availability of Healthcare: The Predominance of Private Centers and the Crisis of Overcrowded Public Centers

As a starting point, it is important to clarify what is meant by private and public health centers in the context of this study. Private health centers include registered private hospitals, clinics, and dispensaries, which operate primarily on a for-profit basis and are known for their high service costs. In this study, private health centers include both formal and informal health centers. On the one hand, formal private healthcare providers have a reputation for offering high-quality, regulated, and reliable medical services. However, their services are often unaffordable, making them inaccessible to a large proportion of the population. On the other hand, this affordability gap has contributed to the rise of informal healthcare providers, such as “street pharmacies” (informal stalls on the pavement that sell medication) and traditional medicine practitioners’ centers where patients will often receive consultation and medication prescription services as well as the sale of medicines. While these informal providers charge relatively lower prices, they operate without regulation, leading to concerns about the quality of service and patient safety. Both formal and informal providers operate within an unregulated pricing system, further complicating the accessibility and affordability of healthcare in Douala.

Public health centers are funded and managed by the government and include public hospitals, health centers, and clinics. These centers are designed to provide affordable and quality health services.

**Figure 5:**
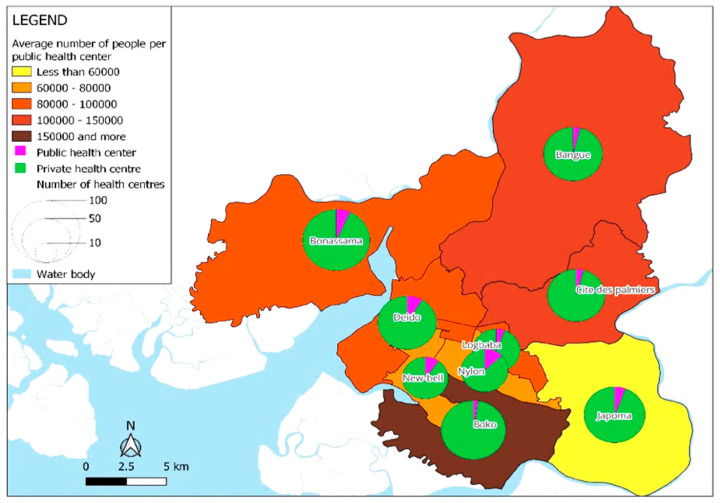
Distribution of the type of healthcare facilities by health districts and the density of population per public health center in the city of Douala (2019).

That said, the distribution of health facilities in Douala is heavily dominated by the private sector, which represents 94% of health facilities, while the public sector provides only 6%. There is approximately one public health center for every 55,245 inhabitants. The healthcare landscape in Douala reflects significant anomalies regarding access to medical care, underpinned by two interrelated challenges: the prominence of a private sector that is characterized by the high cost of medical services, and the overcrowding of the few public health centers, which reflects a critical deficit in healthcare provision by the government (Figure 5).

The map highlights the distribution of healthcare centers in Douala and the population density per public health center across districts. It reveals significant disparities, with some districts, such as Boko and Bangue, which mainly consist of informal settlements that have over 100,000 people highly relying on a few small public health centers of Category 6 or 5 (see Table 1). These areas are marked in brown to dark brown, indicating a severe overcrowding of public facilities. Moreover, private health centers in these underprivileged neighborhoods are mostly small dispensaries providing basic outpatient care or street pharmacies.

In contrast, well-planned districts like Akwa and Bonanjo are better served, with fewer people per public health center. In these wealthy neighborhoods, private hospitals and clinics offer more specialized healthcare services. The pie charts in Figure 5 show the dominance of private healthcare facilities in all districts, which significantly outnumber public facilities.

Applied to the city of Douala, socio-spatial theory helps to explain how historical forces such as colonial land policies, post-independence planning failures, and the increasing informalization of urban growth have collectively produced a deeply unequal urban landscape characterized by a stark contrast between informal settlements and formal neighborhoods. This shows that disadvantaged neighborhoods are not accidental outcomes of demographic growth, but have been structured through historical processes of exclusion, land dispossession, limited state investment, and unregulated expansion. This has led to the formation of socio-social patterns of inequalities in health distribution and accessibility. Given that 94% of healthcare provision in Douala is delivered by the private sector—with both formal and informal providers operating within an unregulated pricing system—a pattern of persistent inequalities is created and perpetuated. Formal private healthcare centers, which are known for offering high-quality, reliable medical services, are typically located in well-planned business districts, and remain unaffordable for most of the population. Meanwhile, private health centers in disadvantaged neighborhoods tend to be small dispensaries that merely provide basic outpatient care. These dispensaries and street pharmacies often lack adequate regulation and quality assurance, reinforcing disparities in health outcomes

#### 3.1.2. Accessibility to Quality Healthcare: Economic and Environmental Challenges

Douala’s urban landscape reflects stark inequalities between planned and unplanned neighborhoods, which significantly impacts accessibility to quality healthcare. Planned areas, mainly in health districts such as Deido, Nylon, Logbaba, and Japoma, are well structured with high- and medium-standard housing, legal land permits, and basic infrastructure. These neighborhoods, which are home to government offices and affluent families, benefit from paved roads, serviced plots, reliable utilities and efficient sewerage, drainage systems, and better-equipped public and private health centers, ensuring better living conditions and medical access.

In contrast, there are unplanned areas, including low-income settlements such as Bangue, and flood-prone, informal settlements such as Boko and Bonasama. These areas lack infrastructure and basic services and face severe environmental risks, such as recurrent flooding. These inequalities contribute to significant public health and infrastructure challenges, particularly in vulnerable, low-income communities. Access to quality healthcare is therefore severely limited due to a combination of poor infrastructure, economic constraints, and reliance on alternative/informal care services. These challenges are discussed below:

- *Environmental and infrastructural challenges of mobility in the event of health emergencies*

**Figure 6:**
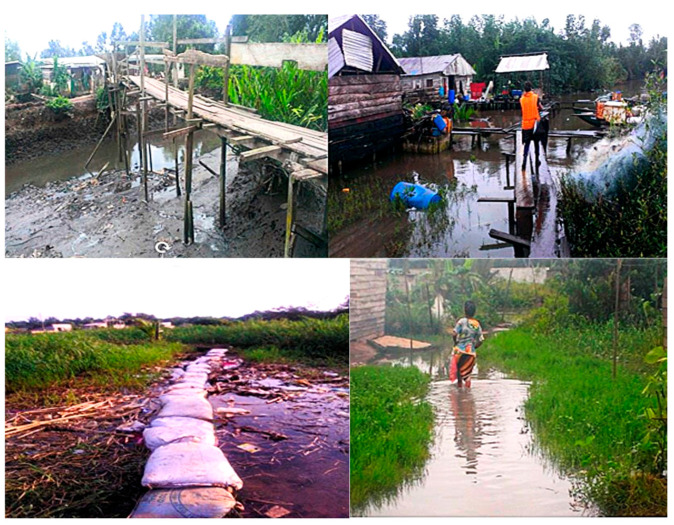
Different types of roads are found in disadvantaged areas: suspended paths, sandbag tracks laid on mud, and flooded roads forcing residents to walk in water. Field survey.

The mobility problems faced daily by residents of disadvantaged neighborhoods significantly limit their access to hospital care. In addition to recurrent flooding, inadequate transport infrastructure is a major risk factor, especially in situations where timely medical intervention is critical. In contrast to Douala’s better-planned neighborhoods, where the roads are laid out and practical for most vehicles, the city’s informal and precarious housing areas are known for their almost non-existent practicable roads. Access to healthcare is, therefore, severely compromised by poor infrastructure. Roads in these areas range from suspended paths and sandbag tracks laid over mud to flooded streets, forcing residents to navigate through water. These difficult conditions considerably increase not only the risks, but also the time and cost of transport to reach health services, particularly in an emergency. Figure 6 illustrates the different types of roads in these neighborhoods, highlighting the mobility problems that residents face when seeking medical care. This is even more critical given that in the rainy season (almost all year round), they are regularly flooded, with all the numerous health risks that this entails.

- *Economic context and affordability: barriers to healthcare access in disadvantaged neighborhoods*

An analysis of healthcare provision in Douala reveals a serious shortage of public facilities offering essential services and care. This shortage is increasingly being made up by private, for-profit health centers, where the cost of care is often very high. Douala’s socio-economic context is marked by major disparities in living standards, which accentuate the vulnerability of low-income households in precarious neighborhoods and significantly affect their ability to access healthcare. The city profile report for Douala states that 72.10% of heads of household do not have stable, well-paid jobs, and they depend on the informal sector to survive. Only 8.1% of the working population of Douala earns between XOF 188,000 and 376,000 (approx. USD 305.69 to 611.38), and only 3% earn over that amount. This economic context accentuates the difficulties of access to healthcare for the majority of the city’s residents.

**Figure 7:**
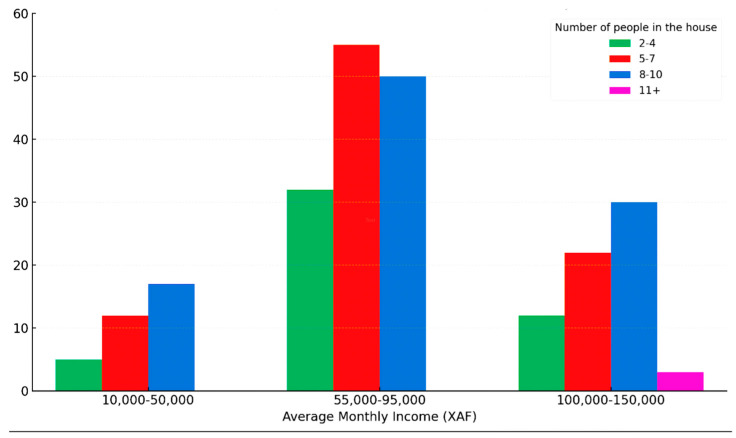
Distribution of average monthly income by household size—field survey.

Moreover, the field survey revealed that 71.4% of surveyed households subsist on average monthly incomes ranging between USD 45 and 192 (XAF 28,500–120,000). The low average monthly income of the household head in comparison to household utilities, especially considering the large household sizes, exacerbates the precariousness and vulnerability of these households in terms of health conditions (Figure 7).

The figure illustrates the relationship between average monthly income (in XAF) and household size, revealing the financial strain on larger households with low earnings. The chart indicates that most households earning approximately USD 88–152 (XAF 55,000–95,000) have 5–7 or 8–10 members, placing significant economic pressure on household heads. Similarly, lower-income groups earning around USD 16–80 (XAF 10,000–50,000) also tend to have sizable families, making it difficult to afford quality healthcare and basic utilities, further deepening their socio-economic challenges.

As a result, most households in the low-income neighborhoods surveyed tend either to forego necessary medical care or to delay it due to its high cost, which often leads to a deterioration in health. This has led to an increased reliance on self-medication as well as informal and unregulated treatment.

### 3.2. The Proliferation of Alternative Healthcare: Expansion of Informal Caregiving Suggesting Poor Health Literacy in Douala’s Precarious Neighborhoods

In Douala’s precarious neighborhoods, reliance on alternative healthcare has become increasingly prevalent, mainly driven by the search for affordable treatment options. This trend is exacerbated by the poor health literacy of the population, which shapes perceptions and decision-making regarding healthcare access.

Many residents have a limited understanding of diseases, symptoms, and appropriate treatments, leading to widespread misconceptions about health conditions and how to manage them. This knowledge gap leads individuals to turn to informal health providers, including traditional healers, street pharmacies, and self-medication, as these alternatives are often perceived as more accessible, culturally familiar, and less expensive than formal health services. In addition, mistrust of modern medical institutions, often nurtured by misinformation, previous negative experiences, and perceptions of high treatment costs, reinforces the preference for alternative health solutions. Many people delay seeking professional medical care because they fear expensive hospital bills or doubt the effectiveness of conventional treatments. As a result, illnesses that could have been effectively treated at an early stage often escalate into more serious conditions, leading to more expensive medical care, prolonged suffering, and sometimes preventable deaths.

These indicators of poor population health literacy play a key role in the widespread use of unregulated healthcare options. The following practices were identified:

- *Self-diagnosis and self-medication:*

Self-diagnosis in the context of this study is when a non-professional person identifies an illness that is affecting them or a dependent (child, or another relative dependent). In the study area, this is mostly performed by recognizing and self-diagnosing symptoms on the basis sometimes of personal experience, or in rare cases by researching on the internet. During the fieldwork, we often talked to people who said they do not need to go to hospital for certain illnesses. Here is some discourse: “*everyone knows that if you have fever (rise in temperature) and headaches, it is most certainly malaria*... *if, on top of that, you have diarrhea, for example, we are more likely to conclude on typhoid*.”

Self-medication describes the practice of using medicines without a medical prescription, to treat mild symptoms or more serious illnesses. This practice is common because of the high cost of medical treatment and the availability of over-the-counter or traditional medications. While it is perceived as offering quick access to treatment, and can work well for certain mild illnesses, self-medication can entail major risks, including the inappropriate use of medicines, incorrect self-diagnosis, and inappropriate combinations of drugs.

- *Street pharmacy* refers to the illegal and informal sale of drugs, often outside the regulated framework of official pharmacies. In Douala, these medicines are sold in markets, by itinerant traders, or on street stalls. These products, which are often falsified or poorly conserved, present a major health risk, as they may be either ineffective, expired, incorrectly dosed, or toxic. The use of these street medicines is mainly motivated by their low cost and accessibility, but it potentially exposes the population to serious medical complications.
- *Indigenous medicine* encompasses all practices based on the use of “medicinal” plants and ancestral rituals to treat various illnesses. It is generally practiced by so-called “traditional healers”, herbalists, and spiritual practitioners. This medicine is passed down from generation to generation and remains an alternative or complement to modern medicine for many local people.

Indigenous medicine and street pharmacies have become the primary healthcare options for most residents in Douala’s marginalized neighborhoods, often after undertaking self-diagnosis. In total, 71% of households surveyed reported relying on these alternatives as their main source of treatment, turning to hospitals only when these methods proved ineffective or when their symptoms became too severe to manage. Here is some discourse on the perceived efficiency of these practices: “*sometimes if you’re not sure, you can also explain to the people selling medicine out there and they’ll tell you what is wrong and what to take*... *so you don’t have to do loads of expensive medical tests”; “ if it doesn’t work, you try something else....that’s how we juggle it, otherwise we’d be in hospital every day*...”; “*sometimes it works, sometimes it doesn’t. But even what you receive at the hospital doesn’t always work*”. This scenario intensifies the health crisis in these neighborhoods, not only leading to detrimental health outcomes for the population but also placing additional strain on the overall healthcare system. These alternatives, though more affordable, often lack the oversight of regulation.

Through the lens of the social and environmental determinants of health theory, the study illustrates how low incomes, poor health literacy, and infrastructure deficits systematically expose residents of informal settlements to higher risks of poor health outcomes. Applying the social determinants of the health framework positions this study beyond a clinical perspective and instead addresses health inequities as products of sociopolitical and economic structures. The field observations and household surveys conducted in the marginalized neighborhoods of Douala confirm that systemic weaknesses, rather than simply individual choices, are the main drivers of the public health crises experienced.

### 3.3. Healthcare Demand Illustrates an Environment of Health Crisis and Deteriorating Health

Healthcare demand in the precarious neighborhoods of Douala reflects the more general health crisis and the continuing deterioration of public health. Households in these areas are increasingly vulnerable to environmental diseases, linked to poor infrastructure and lack of access to quality healthcare. This section provides an overview of how this situation impacts health.

#### 3.3.1. Demographic and Environmental Characteristics of Informal Settlements Driving Health Vulnerability

The health vulnerabilities observed in informal settlements largely stem from their demographic and socio-environmental characteristics (Table 2). Factors such as age structure, level of education, household size or even prevalence of informal employment can significantly influence patterns of exposure and access to care.

- *Rapid spatial expansion of Douala and unplanned occupation of flood-prone areas by precarious housing*

Douala’s rapid urban expansion, particularly from the 1980s onwards, is due to a combination of economic pressures, population growth, and migration. As the state withdrew from urban planning in the wake of economic crises, there was little or no control over land use and development. This vacuum allowed informal settlements to flourish, especially as low-income populations, unable to afford the high cost of living in central urban areas, sought affordable housing on the outskirts of the city. These areas, often lacking basic infrastructure and services, have developed organically without formal planning, creating a pattern of spatial segregation where the wealthiest residents occupy planned and serviced areas, and the poor are pushed to the periphery.

**Figure 8:**
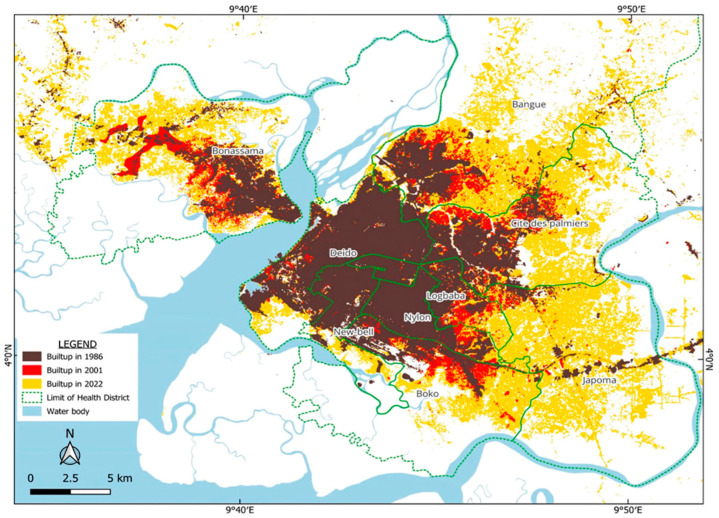
Spatial extension of the built-up area of Douala at different periods (1996, 2001, 2022).

The spatial expansion of built-up areas in Douala has far exceeded the initial sites, which were relatively safe, encroaching on floodplains and areas influenced by tidal movements. This occupation has been unplanned and uncontrolled, which has increased vulnerability to flooding by creating more at-risk sites (Figure 8). This vulnerability is exacerbated by the fact that 80% of homes are built with inadequate construction materials (Figure 8).

- *Lack of appropriate drainage and sanitation facilities*

**Figure 9:**
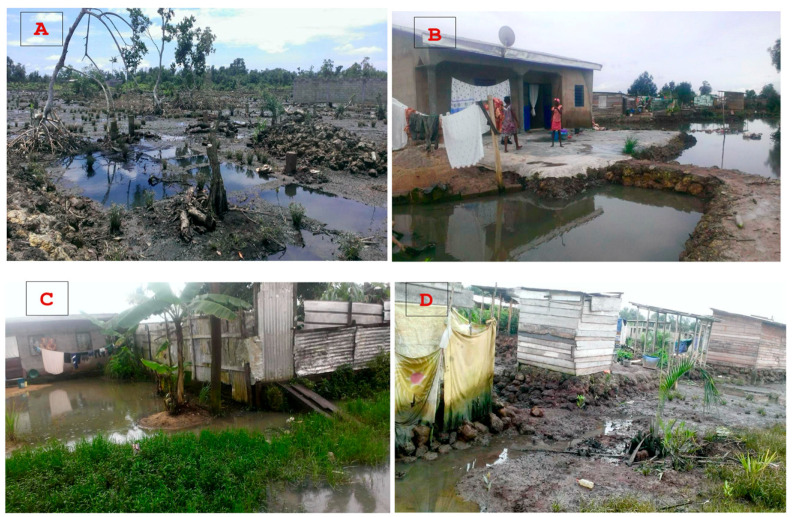
View of the precarious living environment in informal settlements. (**A**,**B**) illustrate the progress of buildings despite environmental constraints. (**C**,**D**) illustrate the poor drainage and sanitation conditions.

The field survey drew attention to the poor state of sanitation in informal settlements (Figure 9). Two key observations emerged. Firstly, open-air latrines are widespread, with 60% of households lacking compliant septic tanks and instead relying on a “natural” emptying process influenced by tidal movements. Secondly, latrines are often constructed using basic materials and are frequently located near water supply points, which increases the risk of contamination. Where septic tanks do exist, they are usually built by unskilled laborers or, in some cases, by the homeowners themselves. Only 20% of households in informal settlements benefit from emptying services provided by specialized vacuum trucks. In certain parts of the Bois de Singe area in the Boko district, some residents live close to the city’s fecal sludge disposal site.

These precarious living conditions characterized by exposure to environmental hazards such as recurrent flooding, water contamination, and inadequate sanitation emerge as key environmental health determinants, leading to increased vulnerability to both infectious and parasitic diseases. Combined with factors such as overcrowded housing and limited sanitation services, these environmental exposures significantly influence health outcomes.

#### 3.3.2. A Healthcare Demand Driven by a High Burden of Disease in Informal Settlements

Based on field observations and surveys, most of the neighborhoods with a high need for healthcare, particularly for diseases related to poor hygiene, are in the Boko, Bonassama, and Bangue health districts. These districts are characterized by a dominance of informal settlements, where limited access to sanitation facilities, non-potable water, and overcrowding contribute to the spread of environmental diseases such as cholera, gastroenteritis, and typhoid.

The field survey conducted in the Boko health district was designed to assess the most common health problems affecting households, how illnesses are diagnosed, which groups are most affected, and the perceived causes of these health issues. The responses of household heads provide critical insights into the health vulnerability of these communities.

**Figure 10:**
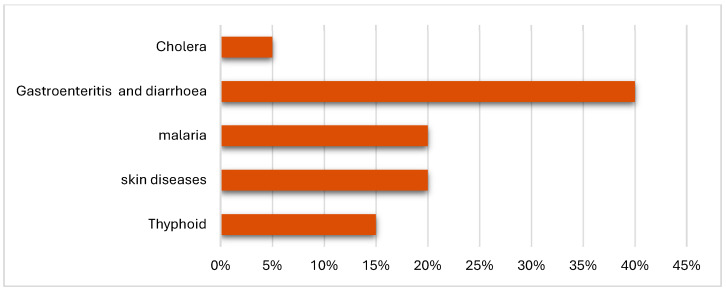
Most common diseases affecting households—field survey.

When asked about the most common diseases or symptoms affecting their households, respondents identified several water-borne and vector-borne diseases (Figure 10). This result emphasizes the impact of environmental factors such as water pollution, poor hygiene, and inadequate waste management.

Regarding how illnesses are diagnosed, 60% of householders surveyed said they relied on self-diagnosis based on symptoms, while only 40% consulted a hospital or health center. This reliance on self-diagnosis indicates that there are barriers to accessing professional healthcare, which can lead to delays or errors in treatment, worsening the health status of those concerned. When asked who is most affected by these diseases, 65% of respondents said that children are the first victims, while 35% reported that both children and adults are affected. This highlights the increased vulnerability of children in these communities, probably due to weaker immune systems and greater exposure to contaminated environments. Finally, when questioned about the suspected causes of these diseases, respondents identified non-potable water from wells and boreholes as an important factor, as well as the high presence of mosquitoes which contribute to the transmission of malaria.

These poor neighborhoods clearly have a high prevalence of environment-related diseases, but they report a relatively low number of patients attending local health centers. The reason is that the lack of well-equipped health centers in these vulnerable areas prevents an adequate medical response, forcing residents to seek treatment outside their districts, usually in health centers in nearby districts. This mismatch between healthcare needs and local medical capacity leads to delayed or no treatment, which in turn increases morbidity. Healthcare needs in these areas are therefore underestimated, as not all cases are reported or treated.

#### 3.3.3. More than Just Access: Why Healthcare Density Alone Cannot Solve the Current Health Crisis in Douala

Spearman correlation analysis provides valuable insights into the relationship between access to healthcare and disease prevalence in Douala (Table 3).

Although the overall pattern does not suggest a consistent or statistically significant correlation between the availability or density of healthcare centers and the prevalence of identified diseases such as malaria, diarrhea, cholera, gastroenteritis, and typhoid, some noteworthy observations emerge. The correlation coefficients are mostly low and sometimes have opposite signs. This suggests that healthcare center density alone is not a dependable indicator of health outcomes and disease prevalence across health districts.

This observation highlights the importance of considering more than healthcare supply when assessing a population’s healthcare needs. It emphasizes the impact of wider socio-environmental factors, such as water quality, sanitation services, housing conditions, waste management, and population density, on disease patterns. In poorly served neighborhoods, for example, a lack of clean water and inadequate hygiene infrastructure can lead to a high prevalence of water-borne diseases, regardless of the number of healthcare centers. Similarly, overcrowding in informal settlements can reduce the effectiveness of health interventions and exacerbate disease transmission, even in areas with adequate infrastructure.

#### 3.3.4. Systemic Inequalities and Spillover of Health Demand to Better-Equipped Districts

Better-equipped public health centers in adjacent districts, such as Deido and Nylon, are increasingly overwhelmed by patients from underserved informal settlements. In particular, the limited capacity and poor infrastructure of both public and private health facilities in districts such as Boko and Bonassama push residents to seek medical care elsewhere. This results in a significant spillover of patients into neighboring districts, putting additional pressure on their health systems. Figure 8 illustrates this dynamic, highlighting the mismatch between areas of high disease burden and the availability of adequate health services. Deido and Nylon record high levels of patients, even though they belong to planned urban areas where exposure to environmental diseases is lower. The influx of patients from health districts with large numbers of informal settlements significantly increases the overall healthcare demand in wealthier districts, overloading public hospitals. The resulting overcrowding leads to longer waiting times, strains medical staff, and reduces healthcare efficiency, ultimately resulting in a decline in the quality of healthcare, even in wealthier areas. This phenomenon exemplifies health inequity, where people in low-income areas must overcome numerous obstacles to access essential care, while well-resourced districts have to cope with service saturation, undermining their ability to provide timely and effective treatment.

The uneven distribution of health centers in Douala contributes to the emergence of a systemic health crisis. On the one hand, informal settlements experience a high burden of disease but lack adequate medical infrastructure. On the other hand, better-planned neighborhoods with relatively well-equipped public health centers are overwhelmed by the influx of patients from the city’s various health districts.

As a result, higher case-fatality rates are observed not only in under-resourced districts such as Boko and Bonassama, but also in the overburdened public centers of Deido, Nylon, and Logbaba. Meanwhile, poorly equipped health facilities in informal areas are unable to meet the high local demand, leading to untreated illness, delays in care, and avoidable complications or deaths.

This situation highlights that the demand for healthcare in Douala is not simply a function of disease prevalence. Rather, it reflects deep-rooted structural inequalities, with wealthier neighborhoods struggling to accommodate an excessive number of patients, while poorer neighborhoods remain underserved, further exacerbating the crisis.

**Figure 11:**
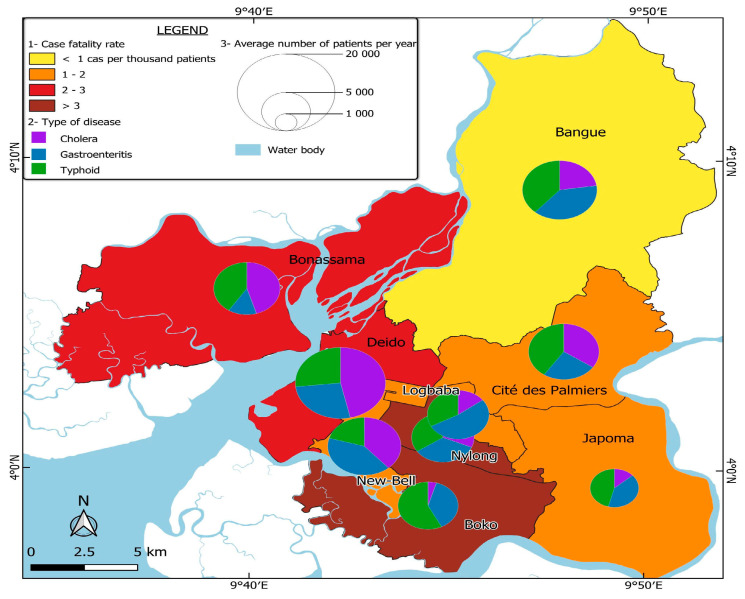
Health demand and evaluation of capacity response for some environmental diseases in Douala Health Districts.

Figure 11 illustrates this reality by providing a spatial assessment of health demand in Douala. It maps the prevalence of three major infectious diseases—cholera, gastroenteritis, and typhoid—across different health districts. The analysis includes case-fatality rates, disease distribution, and the average number of patients, highlighting the stark disparities in health needs and response capacity across the city.

## 4. Discussion

The healthcare challenges in Douala are deeply rooted in the city’s urbanization trajectory, which has produced stark socio-spatial inequalities. From colonial-era segregation to the rise of informal settlements driven by migration to the city and urban planning deficiencies, Douala’s growth has been marked by a persistent imbalance in resource allocation and service accessibility. These patterns of urbanization have directly influenced the distribution and availability of healthcare services, particularly in marginalized neighborhoods. As the city’s population has grown rapidly, healthcare provision has become increasingly dominated by private facilities, which cater primarily to higher-income groups, while public health centers struggle with overcrowding and insufficient resources. In informal settlements, inadequate infrastructure, economic vulnerability, and environmental risks exacerbate the barriers to accessing quality healthcare, reinforcing health inequities. This context highlights how Douala’s urban development process has not only shaped the physical landscape of the city but also deepened inequalities in health outcomes, particularly for its most disadvantaged residents. The results of this study explore these interconnections and their implications for addressing health inequity in African cities such as Douala.

The findings show that health inequities in Douala are not simply a result of rapid urbanization or population growth but rather emerge from deeply embedded socio-spatial structures that can be traced back to colonial land-use policies and post-independence urban planning failures. This reality is not unique to Douala, but reflects more general trends many Africa cities, whereas colonial legacies have significantly shaped the spatial distribution and post-colonial policies have largely failed to reverse it, allowing informal settlements or precarious neighborhoods to expand in vulnerable areas prone to environmental hazards such as flooding. This aligns with a study by Baruah, N. G., Henderson et al. 2020 [27], which examined a sample of 318 African cities and found that institutions inherited from colonization continue to affect the spatial structure and living conditions of millions of people in sub-Saharan Africa.

The use of socio-spatial theory in this study provided a lens to understand how spatial patterns resulting in uneven access to basic infrastructure, services, and opportunities, such as quality healthcare, are produced. It therefore serves to justify the finding that informal settlements have worse physical conditions and inadequate infrastructure, reflecting the socio-spatial inequalities produced over time. A study by Magidimisha-Chipungu and Chipungu (2021) in southern African cities established the same connection [28]. These inequalities are evident in stark differences in living conditions, service provision, and social outcomes across neighborhoods. They often perpetuate cycles of disadvantage, particularly in informal settlements and marginalized areas.

Subsequently, the “social and environmental determinants of health” approach provided a framework for interpreting how living conditions and environmental factors influence residents’ health risks and outcomes. It informed the analysis of the socio-economic and environmental factors that cause health vulnerabilities in informal settlements. This supports the idea that poor living environments and limited public services result in higher disease rates and a greater need for health services. This is consistent with studies that argue those living in informal settlements are often exposed to social and environmental risk factors [29]. Similarly, Emal Ahmad Hussainzad and Zhonghua Gou (2025) [30] demonstrated that demographic characteristics tend to determine health and well-being. For example, they showed that employed and better-educated people presented higher well-being, while lower education levels were strongly related to poor health outcomes.

The dominance of informal settlements in these underprivileges zones—and their vulnerability to environmental hazards—correlates with the higher burden of disease observed, particularly water-borne and vector-borne diseases [4,7,31,32,33]. These findings empirically ground socio-spatial theory in the lived realities of Douala’s residents, aligning with what Soja (1980) described as the socio-spatial dialectic, where urban space is socially produced and reproduces inequality [16].

This study also shows that the health system in Douala is spatially mismatched with the needs of the most vulnerable population. While the city hosts 17% of the national health infrastructure and 76% of that in the region, this quantitative abundance masks a qualitative and spatial deficit. Vulnerable populations must navigate a fragmented health system dominated by unaffordable private providers and under-resourced public centers. That said, this study critically examines the coexistence of urban advantage and urban disadvantage in Douala. Although the city is home to specialized hospitals and economic opportunities, these advantages are unevenly accessible. The study suggests that marginalized communities experience not only restricted access to the perceived benefits of urban life but also increased exposure to environmental hazards, precarious housing, and economic vulnerability. The urban health penalty is therefore manifested as the systematic reproduction of health risks in the urban periphery where informal neighborhoods have become epicenters of persistent health crises.

This study further demonstrates that the socio-spatial dynamics at play in the city of Douala reproduce entrenched inequalities and exacerbate systemic health vulnerabilities through a pronounced spillover effect. The findings show that inadequate healthcare infrastructure in marginalized, informal settlements may contribute to the increase in disease prevalence within these vulnerable communities, driving demand overflow into better-equipped public health centers in planned neighborhoods. This influx of patients puts additional strain on public health centers in planned areas such as Deido and Nylon, resulting in overcrowding, longer waiting times, and a decline in the quality of care. As a result, spatially produced inequalities in quality healthcare provision do not remain confined to underserved neighborhoods, but instead have a wider impact, compromising the resilience and efficiency of the entire city health system.

Furthermore, the research highlights the role of informal health practices in the context of limited public services and the unaffordability of formal private health centers. Residents of low-income neighborhoods have institutionalized informal care through street pharmacies, indigenous medicine, and self-medication as both a response to and a symptom of systemic healthcare deficits and low health literacy. The proliferation of these unregulated practices highlights the adaptive strategies of poor communities but also points to systemic neglect. This is consistent with recent findings by Afeadie (2022) [32] and Cai & Mustapha (2023) [5] that informal care has become a structural response to systemic exclusion, rather than simply a coping mechanism. This dynamic has not been sufficiently explored in the literature, which often treats informal health systems either as marginal or as cultural artifacts, rather than as necessary but risky coping mechanisms arising from structural exclusion. Another study by Conteh et al. (2025) [34] qualifies the practice of health-seeking by people living with non-communicable diseases through informal healthcare providers as a coping mechanism amidst urban inequalities affecting marginalized populations living in informal urban settlements.

Although this study provides valuable insights into the links between urbanization, socio-spatial inequalities and health inequity in fast-growing cities like Douala, a few limitations should be noted. Firstly, the study primarily relies on cross-sectional household survey data and available administrative records, which may not fully capture seasonal or long-term variations in health outcomes and care-seeking behaviors. Secondly, while the spatial analysis does highlight patterns in the distribution and healthcare demand, it is limited by the availability and level of detail in official healthcare center data, which may omit some informal or unregistered healthcare providers. Lastly, the study does not quantitatively model the intricate relationships between environmental factors, social determinants, and health outcomes. This could be an important area for future research. Despite these limitations, the robust empirical basis provided by the mixed-methods approach employed enables a better understanding of how structural urban inequalities contribute to health crises, and highlights the need for more nuanced, context-specific policy interventions.

## 5. Conclusions

This study examined how rapid urbanization, historical legacies, and persistent socio-spatial inequalities intersect to shape health inequalities in Douala, Cameroon. Drawing on socio-spatial theory and the social and environmental determinants of health, as well as the urban advantage/penalty framework, the research highlights that health crises in Douala are not isolated incidents and are not solely a consequence of rapid urban growth. Instead, they are structurally produced and spatially concentrated in disadvantaged neighborhoods.

Our empirical findings suggest that the mismatch between the location of health infrastructure and areas with the most pressing health needs perpetuates unequal access to quality healthcare. This spatial imbalance fuels a spillover effect, whereby residents from underserved, vulnerable neighborhoods overwhelm better-equipped public centers in planned districts. Ultimately, the overcrowding, longer waiting times, and inefficiencies that result compromise the resilience of the entire urban health system, thus illustrating how urban inequality can generate system-wide consequences. Furthermore, the widespread reliance on informal health practices in low-income communities is a critical adaptive response to systemic neglect and unaffordable formal care. While these strategies demonstrate community resilience, they also expose residents to unregulated and potentially harmful treatments, perpetuating cycles of poor health outcomes.

Combined, these insights show that tackling health inequalities in Douala and other rapidly urbanizing African cities requires challenging entrenched socio-spatial structures, improving equitable health planning and incorporating underserved settlements into formal urban frameworks. To reduce dependence on informal care, policy efforts should prioritize expanding affordable, quality healthcare to high-risk areas, improving basic infrastructure, and promoting community health literacy. By addressing these structural drivers, fast-growing African cities like Douala can move toward realizing the potential health benefits of urban living for all residents, rather than perpetuating an urban penalty for the most vulnerable.

## Figures and Tables

**Table 1:** Healthcare pyramid in Cameroon.

Healthcare Categories	Description	Expected Services Provided
Category 1 General and Reference Hospitals	Highest-level healthcare facilities, serving as centers of excellence and reference for the entire health system.	- Specialized and advanced medical care across all disciplines. - Highly advanced diagnostic and therapeutic services. - Cutting-edge surgical interventions. - Teaching and research facilities to train healthcare professionals and develop medical expertise.
Category 2 Central Hospitals	These hospitals also provide a high level of medical care and serve as key referral centers for regional and district hospitals.	- Comprehensive healthcare across a broad spectrum of specialties. - Advanced diagnostic and treatment facilities (e.g., CT scans, MRIs). - Management of severe and complex medical conditions. - Highly specialized surgical and medical procedures.
Category 3 Regional hospitals	Provide advanced medical care and a wider range of specialized services compared to district hospitals.	- All services offered by Category 4 facilities, but with greater capacity and expertise. - Advanced diagnostic and imaging services (e.g., X-rays, ultrasounds). - Comprehensive care for chronic and complicated cases. - A broader range of surgical and medical specialties.
Category 4 District Hospitals	Provide a more comprehensive range of medical services, including some specialized care.	- Internal medicine (e.g., management of chronic illnesses). - Obstetrics and gynecology, including deliveries and basic surgical procedures. - Pediatrics for common childhood illnesses and vaccinations. - Surgery, primarily for routine or non-complex cases.
Category 5 District Medical Centers	A bit larger than integrated health centers and offer both the “minimum package of services” and some additional complementary services.	- All services provided by Category 6 facilities. - Additional diagnostic capabilities, such as simple laboratory tests. - Management of moderately complicated cases that do not require inpatient admission. - Some specialized consultations in basic fields like internal medicine or obstetrics.
Category 6 Integrated Health centers	Provide the most basic level of healthcare services, known as the “minimum package of services.”	- Outpatient consultations for minor illnesses. - Preventive services such as vaccinations and basic health education. - Basic maternal and child health services. - Management of uncomplicated diseases.

**Table 2:** Summary of the demographic characteristics of households in informal settlements.

Demographic Characteristics of Households	Percentage (%)		
**Age**			
≤20	3		
20–30	23		
30–40	34		
40–50	26		
50–60	10		
≥60	4		
Total	100		
**Sex**			
Male	70		
Female	30		
Total	100		
**Matrimonial status**			
Married	90		
Single	5		
Divorce	2		
Widow(er)	3		
Total	100		
**Level of education**			
Never attended school	14		
Primary education	29.6		
Secondary (incomplete)	56.6		
Secondary (completed)	3.9		
Post-secondary/Higher education	3.9		
Total	100		
**Household size**			
2–4	22		
5–7	39		
8–10	38		
>11	1		
Total	100		
**Main activity**	**%**	**Formal/Informal sector**	**%**
Security guard	9	Formal	12
Civil servant	3	Formal	
Fisherman	15	Informal	88
Small business	33	Informal	
Small trade	30	Informal	
Transport operator (motorcycle)	10	Informal	
Total	100	Total	100

**Table 3:** Spearman test results.

	Spearman Correlation Coefficient		
Health Variable	Average Number per Public Healthcare Center	Average Number per Private Healthcare Center	Number of Healthcare Centers/1000 Inhabitants
Malaria_Prev	−0.47	0.1	0.25
Diarrhea_S_Prev	−0.14	0.18	−0.35
Cholera_Prev	−0.5	0.42	0.12
Gastroenterisis_Prev	−0.34	−0.19	0.13
Thyphoid_Prev	−0.22	0.03	0.79

## Data Availability

The datasets generated and/or analyzed during the current study are available from the corresponding author upon reasonable request.
